# Interpretation of Frequency Channel-Based CNN on Depression Identification

**DOI:** 10.3389/fncom.2021.773147

**Published:** 2021-12-27

**Authors:** Hengjin Ke, Cang Cai, Fengqin Wang, Fang Hu, Jiawei Tang, Yuxin Shi

**Affiliations:** ^1^Computer School, Hubei Polytechnic University, Huangshi, China; ^2^Faculty of Artificial Intelligence Education, Central China Normal University, Wuhan, China; ^3^College of Physics and Electronics Science, Hubei Normal University, Huangshi, China; ^4^Department of Clinical Laboratory, Huangshi Central Hospital, Edong Healthcare Group (Affiliated Hospital of Hubei Polytechnic University), Huangshi, China

**Keywords:** convolutional neural network (CNN), interpretation, depression, EEG classification, attention

## Abstract

Online end-to-end electroencephalogram (EEG) classification with high performance can assess the brain status of patients with Major Depression Disabled (MDD) and track their development status in time with minimizing the risk of falling into danger and suicide. However, it remains a grand research challenge due to (1) the embedded intensive noises and the intrinsic non-stationarity determined by the evolution of brain states, (2) the lack of effective decoupling of the complex relationship between neural network and brain state during the attack of brain diseases. This study designs a Frequency Channel-based convolutional neural network (CNN), namely FCCNN, to accurately and quickly identify depression, which fuses the brain rhythm to the attention mechanism of the classifier with aiming at focusing the most important parts of data and improving the classification performance. Furthermore, to understand the complexity of the classifier, this study proposes a calculation method of information entropy based on the affinity propagation (AP) clustering partition to measure the complexity of the classifier acting on each channel or brain region. We perform experiments on depression evaluation to identify healthy and MDD. Results report that the proposed solution can identify MDD with an accuracy of 99±0.08%, the sensitivity of 99.07±0.05%, and specificity of 98.90±0.14%. Furthermore, the experiments on the quantitative interpretation of FCCNN illustrate significant differences between the frontal, left, and right temporal lobes of depression patients and the healthy control group.

## 1. Introduction

More than 350 million people are suffering from depression in the world according to the report of the WHO. The report points out that the suicide rate of depression is about 4.0–10.6%. About twenty hundred thousand people commit suicide due to depression every year. As a result, depression has become the second leading cause of death among people aged 15–29. To this end, online end-to-end electroencephalogram (EEG) classification has gained increasing attention for the capability of monitoring and evaluating the status of brain disorders remotely. That is, accurate evaluation of brain state and timely tracking of its development can minimize the risk of falling into danger and suicide.

Electroencephalogram classification has always been a considerable topic in brain neuroscience research and clinical practice. Most of the traditional work relies on feature extraction, which can reduce dimension and explore the signals of interest (Wiatowski and Bölcskei, 2018). However, in most cases, they are closely correlated to subjects, so their reductions remain theoretically feasible and require expensive manual processing (Myers et al., 2016). Among the feature extraction methods, the sparse non-negative matrix factorization achieved an accuracy of 87.4%, which is higher than non-negative matrix factorization, independent component analysis, principal component analysis, and wavelet transform (Lu and Yin, 2015). As the dominant method of EEG feature extraction, the accuracy of time frequency was 87.5% (Mumtaz et al., 2017). Thus, traditional feature extraction methods need expensive computation while the performance improvement is not as expected.

With the booming of machine learning methods, we introduced the most outstanding work below. Mumtaz et al. (2017) proposed a machine learning method to classify features extracted by wavelet transform of EEG signals and achieve high-performance. To effectively identify the heterogeneous lesions of major depression, a spectrum spatial feature extraction method was proposed. It achieved an average accuracy of 81.23% (ShihCheng et al., 2017). A deep convolutional neural network (CNN) was developed to achieve a high Area Under Curve (AUC) of 0.917 on classifying EEG recordings (van Leeuwen et al., 2019). Gemein et al. (2020) applied a temporal convolutional network to classify pathological and non-pathological on the Temple University Hospital Abnormal EEG Corpus (v2.0.0) and obtained an accuracy of 86%. Recurrent Neural Network (RNN) exhibits great potentials to analyze time-series data regarding functional MRI (fMRI) and EEG data. Recently, a deep sparse RNN model (Wang et al., 2019) was proposed to accurately recognize the brain states across the whole scan session and achieve superior classification performance.

Recently, the attention mechanism (Vaswani et al., 2017) has been widely used in various fields of deep learning tasks such as Nature Language Processing (NLP), image, and speech recognition. Its main idea is to focus on the local information of interest while suppressing other useless information. Understanding of neurotic brain diseases often relies on the intrinsic brain rhythm of neural signals (Fitzgerald and Watson, 2018; Logan and McClung, 2019). Therefore, understanding how to combine the brain rhythm with the attention mechanism of the model is very helpful to improve the performance of the classification model by aiming at focusing on the most considerable parts of the target with different weights on the frequency fluctuations.

Moreover, neural networks play a vital role in Artificial Intelligence (AI), which is one finite interpretable black-box function approximators (Li et al., 2019). However, it is a considerable problem to judge and explain whether the neural network makes correct predictions. The objective AI system can help to (1) make suitable decisions, (2) improve the design of the model, (3) make significant discoveries, and (4) deepen the trust in AI. As a typical example, the system for classifying depression is reasonable when the neural network makes the correct classification by identifying the key features in the brain. On the contrary, although the neural network does not analyze the key feature with the correct fine result, the peripheral factors and even make decisions due to the correct recognition of noise or interference, which leads to the high false-positive and cannot meet the medical requirements. Because of this, it is necessary to decouple the black box by measuring the complex relationship between the key features of the brain regarding channels (brain regions) and the model.

To this end, inspired by attention mechanisms (Vaswani et al., 2017) and time-frequency analysis, we propose a Frequency Channel-based CNN (FCCNN) to identify depression accurately and quickly. It combines the brain rhythm with the attention mechanism of the classifier aiming at focusing on the features of interest. Firstly, a frequency attention structure is constructed to discover features of interest in terms of frequency. The FCCNN then utilizes a lightweight CNN to predict the labels quickly. Moreover, the activation maximization (Hinton et al., 2006) was calculated by information entropy based on the affinity propagation (AP) clustering partition aiming at interpreting the FCCNN. The main contributions of this study are summarized below:

1. A frequency attention structure is proposed. With this structure, classifiers can combine the brain rhythm into the attention mechanism of the model and discover features of interest in terms of frequency. Especially for the tensor that contains complex low-frequency fluctuations, it can improve the accuracy.
2. The information entropy based on the AP clustering partition is calculated to measure the activation maximization of FCCNN. It learns the data distribution rather than just assuming that the data obey a uniform distribution. The lower mean entropy values in the regions regarding left temporal and right temporal, frontal lobe conclude that significant differences existed in these brain regions, which reproduced the previous study (Mumtaz et al., 2017).
3. One whole solution has been developed to identify Major Depression Disabled (MDD) subjects. The performance of this solution is overwhelmingly higher than the state-of-the-art methods.

## 2. Methodology

This section details the design and operation of the classifier (see section 2.1) and the interpretation of the classifier on depression identification (see section 2.2).

### 2.1. Design and Operation of Classifier

Electroencephalogram identification in this study is a binary classification problem to recognize one EEG segment whether it belongs to Depression (label: 1) or Healthy (label: 0). A multivariate series (one matrix) *X*^*m* × *n*^ (20 × 1024 in this study) is reshaped into a 3D tensor *T*^*m* × *a* × *b*^ (20 × 32 × 32 in this study) for the input of the FCCNN.

**Figure 1:**
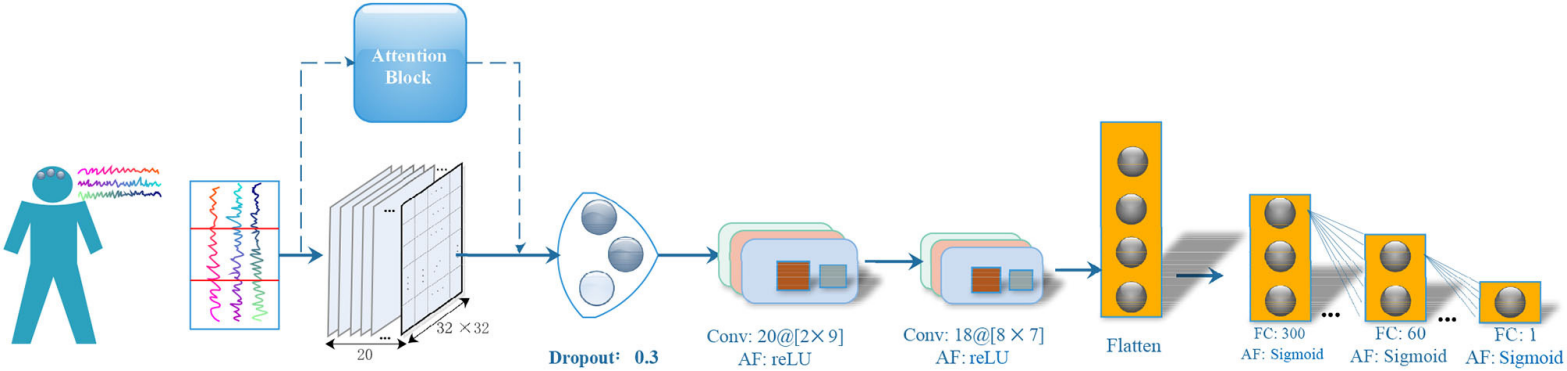
Neural Architecture of FCCNN. “FC” denotes the fully connected layer, “AF” denotes the activation function. The hyper-parameter of convolutional layer is denoted as “filters @ [receptive map size]”.

Figure 1 illustrates the architecture of FCCNN. The main design strategy of the classifier is to use as few network layers as possible without reducing the classification performance. The classifier firstly applied an attention block on the input EEG segment (reshaped to 20 × 32 × 32). It is then followed by one dropout layer, two convolutional layers, one flatten layer, and three fully connected layers. The hyper-parameters of the FCCNN are fine-tuned by our previous grouping Bayesian optimization algorithm (Ke et al., 2020b) and also illustrated in Figure 1. The hyper-parameter of convolutional layer is denoted as “filters @ [receptive map size].” The activation function of all fully connected (FC) layers is “sigmoid,” and that of the convolutional layer is “ReLU.” The final “sigmoid” of FCCNN outputs the classification label of a specific segment. The main design principles are as follows:

- Attention block focuses on the most considerable parts of the target with different weights on the frequency fluctuations. It first extracts the frequency components of each channel according to the FFT algorithm and then calculates the average power of the frequency components. The power values of all channels are normalized to (0.1, 1) and then mapped to the amplitude of the channel as weights.
- FCCNN accepts EEG segments from different channels to extract space features of EEG.
- Fully connected layers play the role of “classifier” to classify the state of the segment in terms of mapping the features learned by previous convolutional layers to the sample tag space.

We use the momentum SGD algorithm with a learning rate of 0.01 to optimize the FCCNN via backpropagation algorithm (Krizhevsky et al., 2012). This study sets a small momentum attenuation factor (decay = 1e-4, momentum = 0.9, nesterov = True) to reduce the residual error (Krizhevsky et al., 2012). The initialization strategy follows the setting in reference (He et al., 2015) and sets the batch normalization of 80 and epochs of 83. The model reports the performance on the test set (or new EEG segment) after training.

### 2.2. Interpretation of the Classifier Based on AP Clustering

This subsection mainly discusses the activation maximization (see section 2.2.1) of the input layer. The feature visualization (Zeiler and Fergus, 2014) of neurons will provide a global view of the network. The network rarely uses neurons in isolation, while understanding stays at the subjective level. To verify the rationality of the model and enhance the objectivity of the interpretation, the information entropy of the input based on AP clustering (see section 2.2.3) is then measured.

#### 2.2.1. Activation Maximization of the Classifier

Activation maximization finds the input mode with the maximum activation value of a given hidden layer unit. The activation function of each node in the first layer is a linear function of the input and proportional to the filter itself. Formally,


(1) $$\begin{array}{l} {\text{x}}^{*}=\arg\underset{\text{x}:\text{t},||\text{x}||=\rho }{\max}\left(h_{ij}\left(\theta ,\text{x}\right)-\lambda \left(x\right)\right) \end{array}$$

where θ is the model parameter of FCCNN, *h*_*ij*_ is the joint function of input *X* and model parameter θ, *h*_*ij*_(θ, *x*) denotes the activation value of the *i*-th neuron in the *j* layer of a neural network, and λ(*x*) is the regular term of input *X* and x^*^ is the maximum activation need to be obtained. Activation Maximization is a non-convex problem in most cases because *h* is a general function. Based on the gradient descent method, the problem can be solved approximately with the local minimum can be solved at least. The gradient of *h* is calculated and *x* along the gradient is moved:


(2) $$\begin{array}{l} \frac{\partial h_{ij}\left(\theta ,x\right)-\lambda \left(x\right)}{\partial x} \end{array}$$

When the amount of moving *x* is less than a predefined threshold, the algorithm converges. Since the input (the first layer) of the classifier is channel-based, we calculate the activation maximization of the first layer to characterize the activation mode of the neural network. In this way, the activation maximization of the first layer is 20 (the same as channel number) activation matrices with respect to the size of the input layer (20 × 32 × 32), each of which represents the maximum activation feature of each channel.

#### 2.2.2. AP Clustering Algorithm

Affinity Propagation clustering (Frey and Dueck, 2007) is a clustering algorithm based on information transfer between data points. The input of the AP clustering algorithm is the similarity (s[i, j] s.t. i,j = 1,2,⋯ , N) between sample data, such as the Euclidean distance. The reference matrix *P* consists of the elements on the diagonal of *S* and represents the probability of each center. The alternating update for the responsibilities matrix R(i, k) and the availability matrix A(i, k) is given below:


(3) $$\begin{array}{l} \begin{array}{l} R\left(i,k\right)\leftarrow S\left(i,k\right)-\max_{k^{\prime }s_{s.t.k^{\prime }\pm k}}\left\{A\left(i,k^{\prime }\right)+S\left(i,k^{\prime }\right)\right\}\\ A\left(i,k\right)\leftarrow \min\left\{0,R\left(k,k\right)+\sum_{i^{\prime }s_{s.t.i}^{\prime }\notin \left(i,k\right)}\max\left\{0,R\left(i^{\prime },k\right)\right\}\right\} \end{array} \end{array}$$

Finally, the algorithm finds the cluster center until its convergence.

#### 2.2.3. Information Entropy Based on AP Clustering Partition

Information entropy describes the uncertainty and complexity of information hidden in the data:


(4) $$\begin{array}{l} H\left(X\right)=-\underset{x\in X}{\sum }p\left(x\right)\log_{2}p\left(x\right) \end{array}$$

For each neural data *X*, we calculate the information entropy based on the AP clustering partition as below. First, the *X* is sorted (ascending) to accelerate the convergence speed of AP clustering. Second, applying AP algorithm on the *X* to get the corresponding partitions with the maximum ($Z_{max}^{i}$) and minimum ($Z_{min}^{i}$) coordinates of each partition *i*. The partition center *C*^*i*^ and corresponding partition radius *R*^*i*^ can be then calculated as follows:


(5) $$\begin{array}{l} C^{i}=\frac{Z_{\max}^{i}+Z_{\min}^{i}}{2},R^{i}=\frac{\left|Z_{\max}^{i}-Z_{\min}^{i}\right|}{2},s.t.Z\in X \end{array}$$

Then, we calculate the dividing point between two adjacent partitions *P*^*i*^ and *P*^*j*^ as follows:


(6) $$\begin{array}{l} D\left(i,j\right)=\frac{\left(C^{j}-R^{j}\right)-\left(C^{i}+R^{i}\right)}{2},s.t.|i-j|=1. \end{array}$$

Finally, the corresponding probability of the data falling into different partitions is calculated to obtain the information entropy of *X*. The activation matrix of each channel is obtained (see section 2.2.1) to describe the complexity between the brain state and the classifier. After all the matrices are flattened into series separately, the algorithm will calculate the information entropy based on the AP clustering partition. It then projects the entropy onto a 3D scalp topographies map at the channel level. Furthermore, we visualize the average features of the brain state corresponding to brain regions in terms of 10 to 20 international systems. Table 1 represents the relationship between the brain region and the channels.

**Table 1:** Brain Region based on 10-20 international electroencephalogram (EEG) system.

**ID**	**Region**	**Electrodes**
1	Frontal lobe	Fp1, Fp2, F3, F4
2	Left temporal	F7, T3, T5
3	Central	C3, C4, Fz, Cz, Pz
4	Right temporal	F8, T4, T6
5	Occipital lobe	P3, P4, O1, O2

## 3. Results

We conducted the experiments to evaluate the performance of the proposed approach upon one public available EEG data set of MDD (section 3.1), which consisted of (1) a performance study for MDD identification (section 3.2); (2) an experiment on the interpretation of classifier (section 3.3); and (3) an experiment on the analysis of attention block (section 3.4).

### 3.1. Experimental Setup

#### 3.1.1. Data Description

All samples of 34 MDD patients and 30 Healthy Controls (MPHC, Mumtaz et al., 2017) were collected from the hospital of University Sains Malaysia. MDD participants (17 men, mean age = 40.3± 12.9) with psychiatric symptoms, pregnant women, alcoholics, smokers, and epileptics were excluded from the samples. The healthy control group (21 men, mean age = 38.227 ± 15.64) also excluded possible mental or physical illness. Furthermore, the EEG data were digitized with 256 samples per second, band pass filtered from 0.1 to 70 Hz with an additional 50 Hz notch filter to suppress power line noise. For more detailed information please refer to Mumtaz et al. (2017). Overfitting would occur when performing classification based on subjects. Thus, this study applied time window technology to obtain enough samples. It split all EEG data into 18,442 segments regarding 9,789 MMD and 8,653 Healthy via the time window of 1,024 (4 s). The whole sample space would then be spitted into the training set and test set. Details were available in Table 2.

**Table 2:** The details of training set and test set. HG denotes the health control group and MG denotes the MDD's group.

**Training subjects**	**Training samples**	**Test subjects**	**Test samples**
HG:24 MG:27	HG:6898 MG: 7816	HG:6 MG:7	HG: 1755 MG: 1973

#### 3.1.2. Baselines

On the same data set (MPHC EEG data), different classifiers were utilized to classify the depression, and Table 3 reported the performance indexes. Among these classifiers, except the MLRW (Mumtaz et al., 2017), this study rebuilt several representative neural networks including Resnet-16 (He et al., 2016), GoogLeNet (Szegedy et al., 2015), and Lenet (Lecun et al., 1998). Moreover, we also evaluated our classifier without the attention block. We modified the input as 20*32*32 for all classifiers and output shapes of the models, but other configurations about the layers and hyper-parameters.

**Table 3:** Comparison of different classifiers. The value in brackets represents the SD.

**Approaches**	**Accuracy**	**Sensitivity**	**Specificity**	**Time**
	**(%)**	**(%)**	**(%)**	**(min)**
MLRW (Mumtaz et al., 2017)	87.50	95	80	-
LeNet (Lecun et al., 1998)	93.31 (6.24)	91.93 (4.27)	94.85 (1.81)	2.8
Resnet-16 (He et al., 2016)	82.26 (7.59)	88.90 (2.14)	74.79 (3.83)	80
GoogLeNet (Szegedy et al., 2015)	93.74 (3.65)	96.48 (1.23)	90.62 (4.62)	42
Ours-withoutAttention	96.04 (3.02)	97.75 (2.09)	94.12 (3.58)	3
Ours	99 (0.08)	99.07 (0.05)	98.90 (0.14)	3.5

#### 3.1.3. Training of the Classifier

After all samples in the training set were shuffled and split into training samples (80%) and validation samples (20%), a five-fold cross-validation strategy was adopted for hyper-parameter tuning of the classifier (a total of 20 iterations). Then, the trained model is applied to the test set, and the average performance of the classifier was reported according to its sensitivity, specificity, and accuracy (Ke et al., 2018).

Finally, we calculated the activation maximization of the input to the trained classifier to interpret the FCCNN (see section 3.3).

### 3.2. Performance Study on MDD Identification

This set of experiments evaluated the classification performance in terms of a learning curve, receiver operating characteristic curve (ROC) curve, and performance indexes regarding sensitivity, specificity, and accuracy.

**Figure 2:**
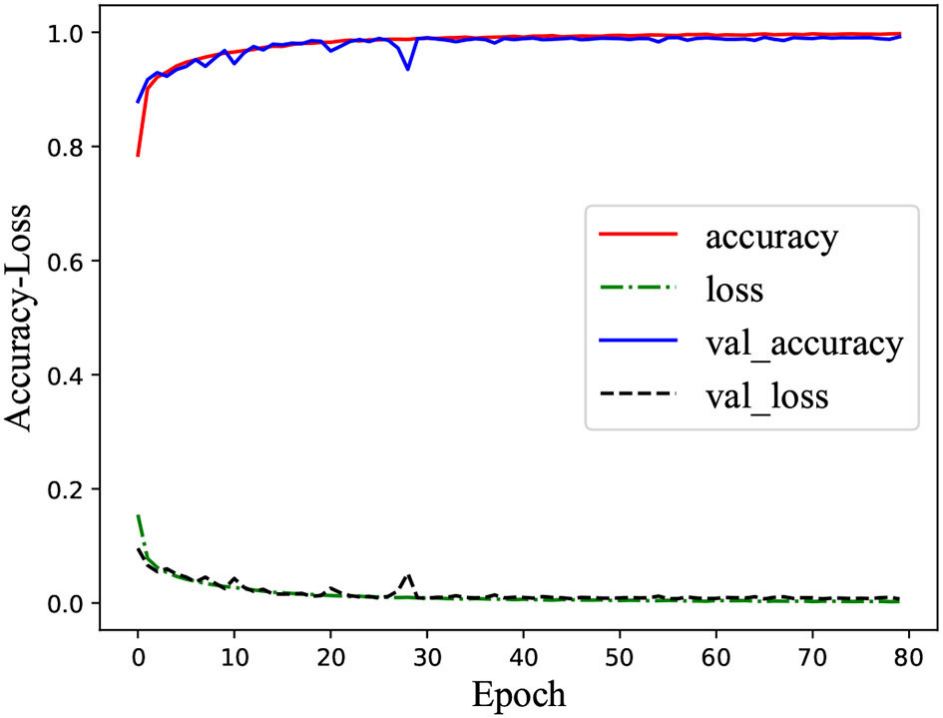
Accuracy and loss rates in the training and validating processes upon MPHC.

Figure 2 was the learning curve of the classifier on training the MPHC data set. Here, “accuracy” and “loss” denoted the accuracy and error in the training stage, respectively; “val_accuracy” and “val_loss” indicated the accuracy and error in the validation stage, respectively. It could verify the generalization ability of the classifier. In the training stage, the accuracy of the classifier on the training set and validation set was consistent, and no obvious gap between the curves existed. At the same time, the excellent classification performance on the test set denoted that the classifier had a desirable generalization ability in the current case study, and the overfitting or underfitting did not occur (Ke et al., 2020a).

**Figure 3:**
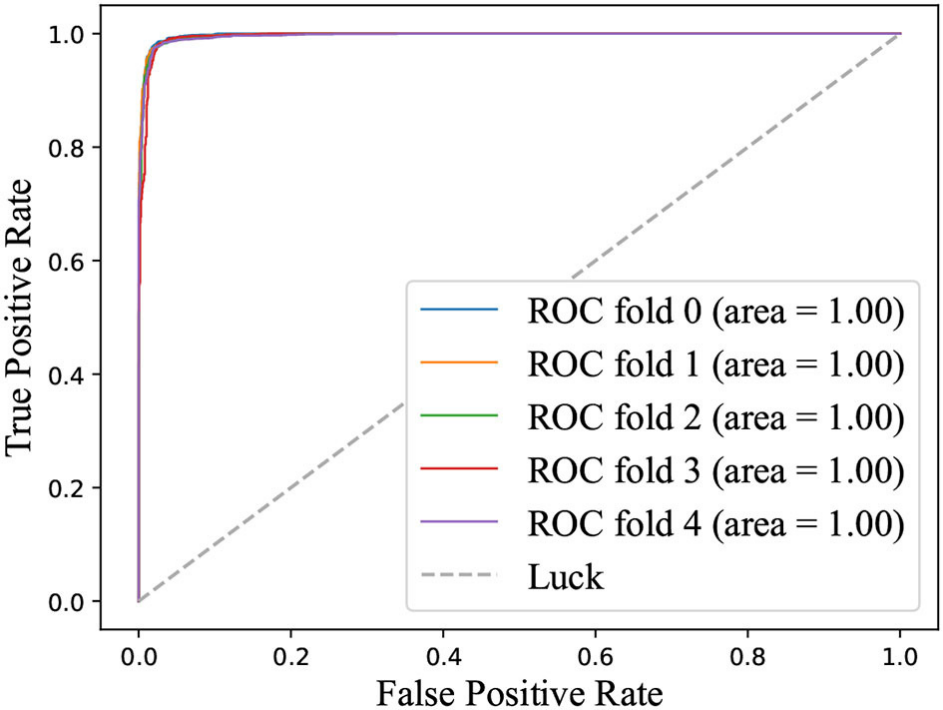
ROC Curve on identifying depression state on MPHC.

ROC curve is introduced into the field of machine learning to evaluate the results of classification and detection. When the positive and negative samples are not balanced, the ROC curve (AUC value) will be a more stable indicator to reflect the quality of the model than the Precision-Recall curve. Figure 3 illustrated the ROC curve on identifying depression state on the MPHC data set. The high AUC (value = 1) indicated that the proposed classifier could distinguish the depression state effectively.

The table represented that the classifier proposed in this study was the best in all performance indicators. Meanwhile, the attention block could improve the performance and stability with lower SD, and high sensitivity and specificity also illustrated that the classifier could effectively screen out patients with depression and health controls together.

**Figure 4:**
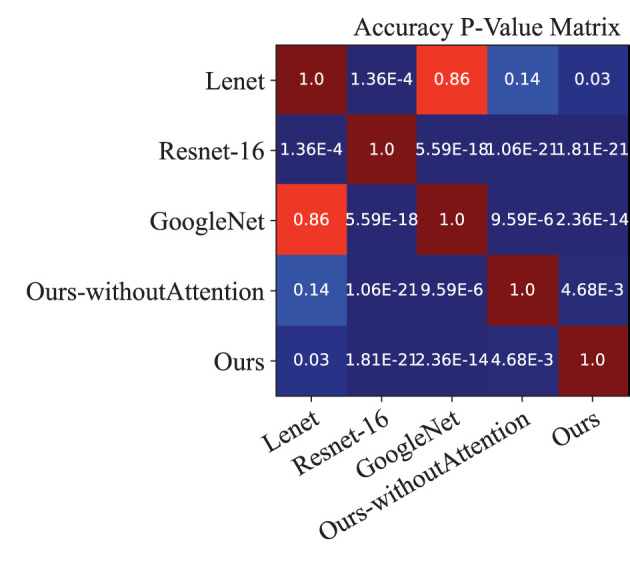
*p*-value matrix on performance index of accuracy.

**Figure 5:**
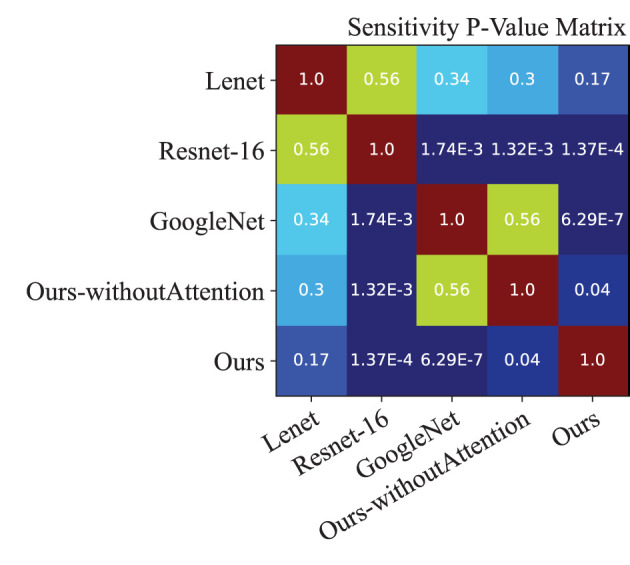
*p*-value matrix on performance index of Sensitivity.

**Figure 6:**
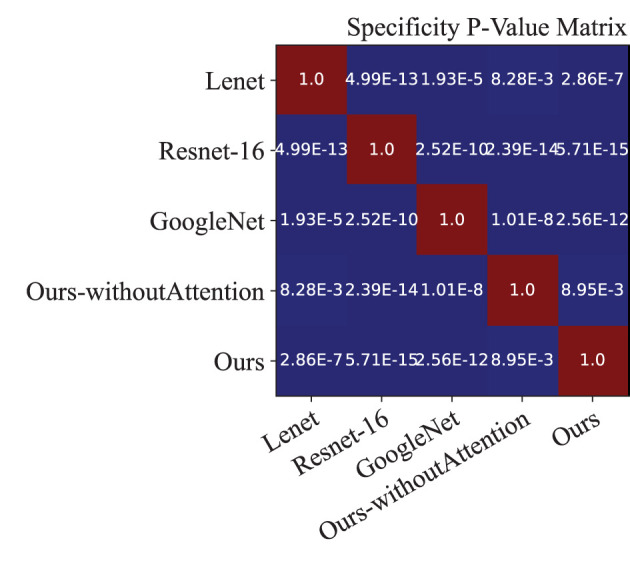
*p*-value matrix on performance index of Specificity.

Moreover, we performed a *t*-test for most of the approaches on the performance indexes regarding sensitivity, specificity, and accuracy to evaluate discrimination via *p*-values. Figures 4–6 illustrated the *p*-values on performance indexes regarding accuracy, sensitivity, and specificity, respectively. From the figures, we concluded that (1) greater statistical significance of most approaches were observed for the three performance indexes (cool color), (2) all the *p*-values on specificity illustrated statistical significance, (3) smaller statistical significance between Lenet and other approaches on accuracy and sensitivity were observed (hot color), and (4) the *p*-values on the diagonal of the matrix did not make sense.

### 3.3. Interpretation of FCCNN on Identifying MDD

This set of experiments was to explain the FCCNN on identifying MDD. The classifier always tended to classify according to the features with significant differences (less complexity) in the classification problem. In the field of information, entropy was a measure of the uncertainty on random variables. To our best knowledge, the greater the information entropy, the greater the amount of information contained in the variable, and the greater the uncertainty of that. In summary, the classification was that of reducing uncertainty (complexity) of the problem aiming to obtain lower entropy.

Moreover, the activation maximization of the input layer of the classifier was visualized to understand the mechanism of the classifier in processing EEG data because the input of FCCNN reflected the channel level characteristics of EEG data. The activation maximization of the first layer is 20 (the same as channel number) activation matrices with respect to the size of the input layer (20 × 32 × 32), each of which represents the maximum activation feature of each channel. Then, the AP clustering partition algorithm calculated the information entropy of each activation matrix and projected it to the scalp topographic map at the channel level. Besides, we also visualized the average entropy corresponding to brain regions partitioned by Table 1.

**Figure 7:**
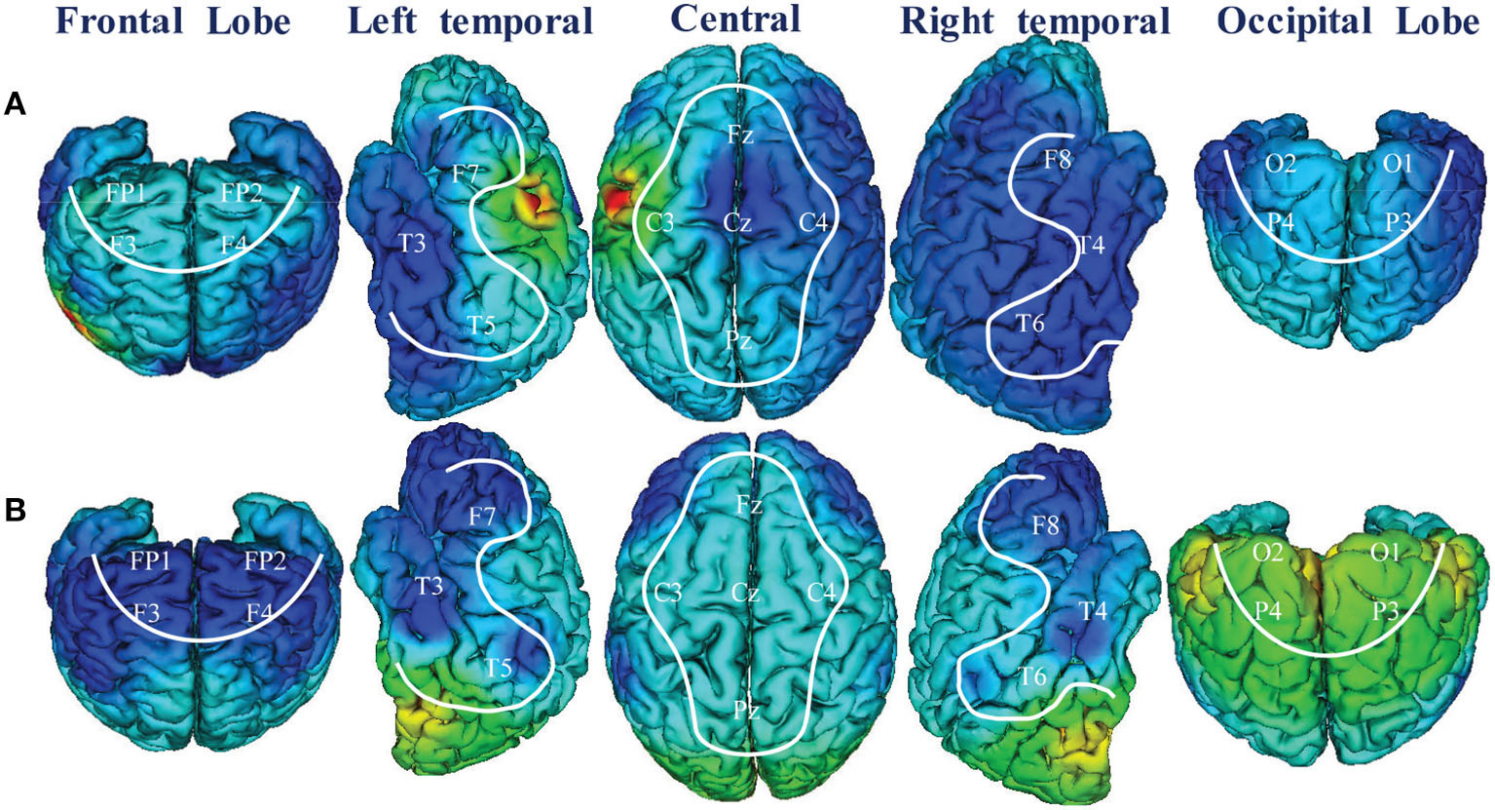
3D scalp topographies map visualizations from FCCNN with channel **(A)** and brain region **(B)**.

Figure 7 illustrated the 3D scalp topographies map visualizations from the activation maximization of FCCNN with channel level (Figure 7A) and brain region level (Figure 7B). From Figure 7A, the entropies of channels (Cz, P6, Fp2, F3, F4, O1, O2, F8) were lower than those of other channels, which indicated that the classifier mainly distinguishes depression and health according to extracting and classifying the features hidden in these channels correctly. Figure 7B also illustrated a 3D scalp topographic map corresponding to the brain region level. The lower mean entropy values in the left and right temporal, frontal lobe concluded that significant differences existed in these brain regions. This result reproduced the study of the data provider (Mumtaz et al., 2017), which meant that our model made correct classification by analyzing the key features among the depression state.

### 3.4. Analysis of Attention Block

**Figure 8:**
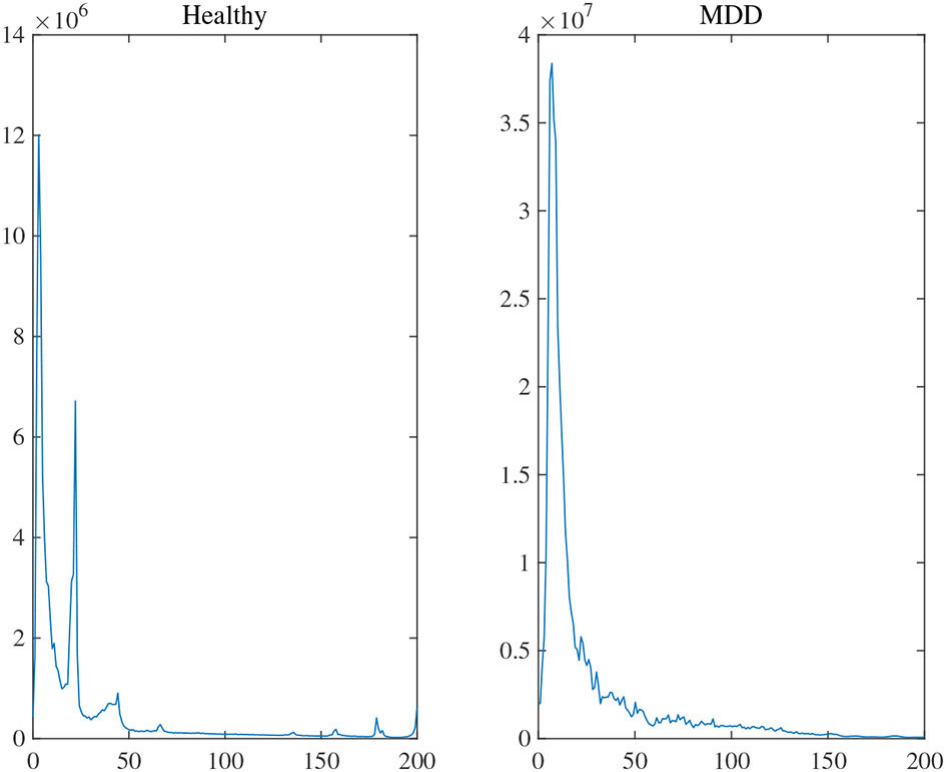
The average frequency-power representations of a different class label (Healthy & MDD) of a typical channel (Fz).

Two experiments analyzed the attention block. The average power [0, 200 Hz] of each channel was first obtained by Fourier transform to evaluate the statistical difference in frequency between MG (segments: 9,789) and HG (segments: 8,653). Notice that the attention used in the training stage was the average power of frequency on each channel. Figure 8 illustrated the average frequency-power representations of the different class labels (Healthy & MDD) of a typical channel (Fz), and similar results were obtained in other channels. From the figure, we arrive at the following conclusions: (1) the frequency distribution was concentrated in low-frequency bands, (2) the power peak of the HG was at 3.015 and 22.11 Hz, while that of the MG was at 7.035 Hz, and (3) the power value of MG was generally higher than that of HG.

**Figure 9:**
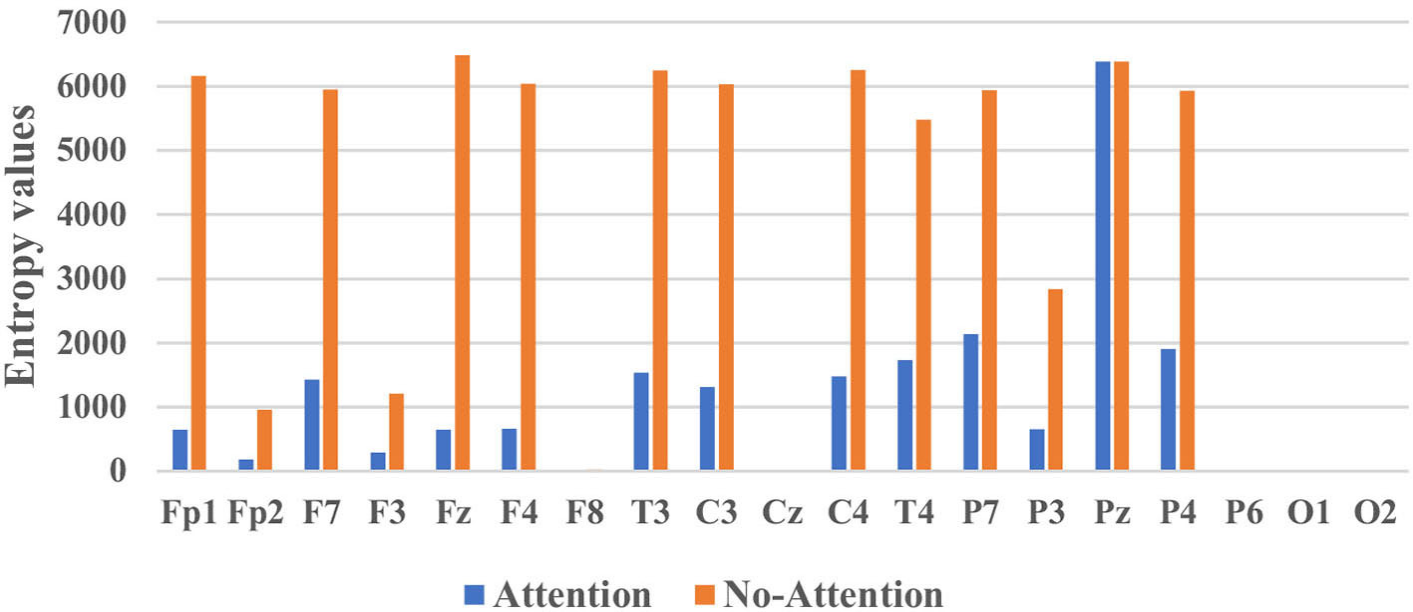
The mean entropy values with and without the attention module.

Figure 9 illustrated the mean entropy values with and without the attention module. They evaluated whether the classifier focused on those “important” channels of interest, especially the channels located in the brain regions of the left and right temporal, frontal lobe (Mumtaz et al., 2017). From Figure 9, we concluded the following insights. First, the information entropy of almost all channels decreased except for Pz, which meant the attention module could (1) greatly reduce the complexity of the classifier, (2) improve the classification performance. The root cause might be that the classifier paid attention to and acted on the features of more channels. Second, the information entropy of channels including F8, Cz, P6, O1, and O2 was very small whether the classifier contained an attention module or not, which meant the classifier with or without attention module were both effective.

## 4. Discussion

First, this section analyzed the computational complexity of the proposed method (see section 4.1). Second, the influence of data partition on a calculation of information entropy was discussed in section 4.2. Third, the influence of Neural Network layers (see section 4.3) and optimizers on performance (see section 4.4) were discussed in detail. Finally, the disadvantages and future research directions of this study were also provided.

### 4.1. Computational Complexity

Experiments were performed on the same Desktop (equipped with AMD R7 3700X CPU@3.59GHz, NVidia RTX 3080 10G GPU, and 16GB RAM on 64bit Windows 7). The classifier proposed in this study was based on a sub CNN and sub dense neural network. The time complexity of the sub CNN was proportional to the number of layers (L) and the corresponding number of neurons (N). Thus, the time complexity was calculated as follows:


(7) $$\begin{array}{l} O\left(S\left(N,L\right)\right)=O\left(\overset{d}{\underset{L=1}{\sum }}n_{L-1}∙s_{L}^{2}•n_{L}\cdot m_{L}^{2}\right) \end{array}$$

where *n*_*L*_ and *n*_*L*−1_ were, respectively, the number of filters (also known as “width”) in the *L*-th and (L-1)-th layers, with the overall network depth d; moreover, *s*_*L*_ and *m*_*L*_ represented the spatial sizes of the filter and the corresponding feature map, respectively.

For the sub dense network, let *L* denote the number of layers and *U* denoted the number of neurons in each layer, the time complexity is *O*(*UL*). In summary, the overall complexity of the proposed approach is *O*(*S*(*N, L*)) + *O*(*UL*).

### 4.2. The Influence of Data Partition on Calculation of Information Entropy

**Figure 10:**
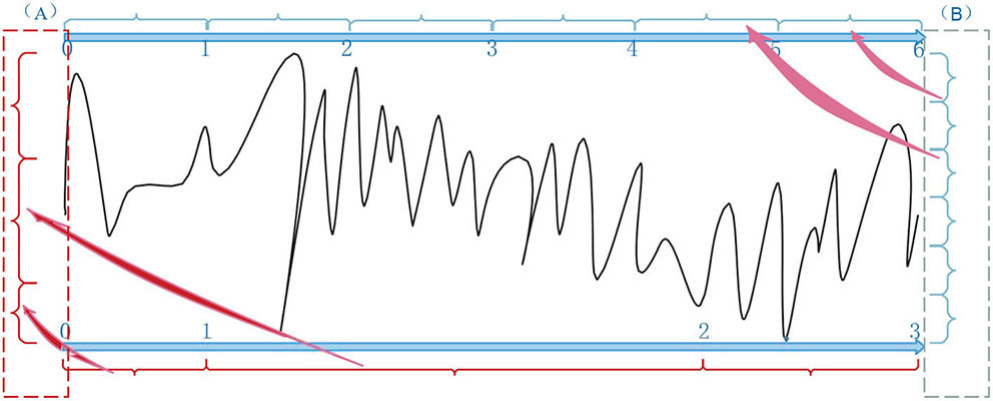
Entropy calculation between AP-based clustering partition **(A)** and traditional methods **(B)**.

Figure 10 illustrated the information entropies according to traditional and our strategy in terms of the data partition. The main difference between the two strategies was the assumptions of data distribution. First, the traditional one divided the neural data into partitions with the equivalent range because of obeying uniform distribution (in Figure 10B, the data was divided into six partitions equally). In this case, the result would be close to the grand truth with enough sample points. However, this would produce a big residual with the insufficient data, which led to the inaccuracy of uncertainty measurement between the model and neural data. Second, our strategy assumed that the neural data obeyed general adaptive distribution. That is, it learned the distribution of the data itself in terms of a data-driven approach and made a reasonable partition (in Figure 10A, the three partitions with different data distribution had obtained). In this case, the algorithm calculated the information entropy accurately and measured the uncertainty correctly.

### 4.3. The Influence of Neural Network Layers on Performance

The classification performance was not related to the number of layers of its model in this study. For example, Resnet-16 and Capsulenet, which had more layers, did not achieve the expected performance indicators but needed a longer training time. The root cause was that the complex classifier might bring the over-fitting problem, which led to the degradation of classification performance. It was a considerable challenge to make the classifier better fit the non-linearity of different data. Furthermore, understanding the non-linear fitting mechanism would be one of the key issues in understanding the neural network black box, which would be one of the key research directions in the future.

### 4.4. The Influence of Optimizer on Performance

**Figure 11:**
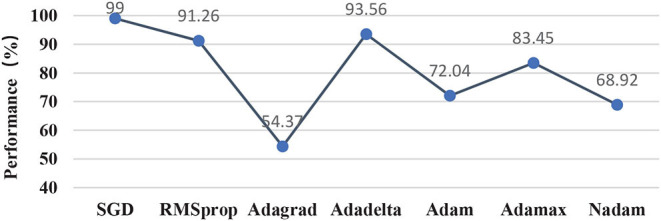
Comparison of candidate optimization methods.

This subsection compared different optimization methods in the classifier, including momentum SGD in this study, RMSprop, Adagrad, Adadelta, Adam, Adamax, and Nadam. Figure 11 illustrated that momentum SGD achieved the best performance, while the three-optimizer including Adagrad, Adam, and Nadam performed poorly in this study. Adagrad optimizer was to modify the learning rate for each parameter according to the previously calculated parameter gradient in each time step. However, the learning rate was always decreasing and decaying, and the learning ability of the model decreased rapidly. In this case, it was very likely that the classification performance became poor without crossing the local minimum value. As an extension of the Adagrad, Adadelta solves the attenuation problem of learning rate and improved performance. Momentum-based methods such as the momentum SGD utilized in this study and the RMSprop optimization method could skip the local optimum. Aiming at training the neural network with complex structure quickly, the optimization methods regarding Adam, Adamax, and Nadam failed to train the light-weighted neural network, such as the classifier in this study. The most likely root reason might be that the oscillation would occur when closing to the optimization goal, which resulted in the performance failing to meet the requirements.

### 4.5. Future Work

It was believed that the extended model should be proposed in the future to enable multiple classifications to identify the subtypes of depression state. Moreover, it played a vitally important role in understanding the dynamic evolution mechanism of multi-dimensional EEG data via interpreting the complexity of the classifier evolution over time. A suitable way in the future to extend the ability of neural networks for processing ongoing EEG data would be the Long Short-Term Memory neural network and temporal CNN (Chen et al., 2020).

The use of a single dataset means that the results should not be generalized to a wider population. In future work, multiple datasets will be created and used for validation of the method.

## 5. Conclusions

The proposed method can achieve high classification accuracy on the public EEG data set of major depressive disorder. Depression is identified with 99±0.08% of accuracy, 99.07±0.05% of sensitivity, and 98.90±0.14% of specificity, which is better than the classification performance of existing methods (based on the same data set). In addition, the information entropy based on the AP clustering partition was utilized to measure the complexity of FCCNN in terms of depression identification. The smaller information entropy of the left temporal lobe, right temporal lobe, and frontal lobe indicates that the FCCNN in this study can correctly identify the intrinsic features of these brain regions. The consistency with the conclusion of the data provider shows the rationality of the proposed approach.

## Data Availability Statement

The original contributions presented in the study are included in the article/supplementary material, further inquiries can be directed to the corresponding authors.

## Author Contributions

FW and HK contributed to the conception of the study and contributed reagents, materials, and analysis tools. CC and FH conceived and designed the experiments. FW, JT, and YS performed the experiments. HK analyzed the data. All authors contributed to the article and approved the submitted version.

## Funding

This work was supported by the grants from the key project of the scientific research program of Hubei Provincial Department of Education (D20214503), the Talent introduction project of Hubei Polytechnic University (21xjz16R, 2019A02), and Scientific research funding project for young teachers of Hubei Normal University (HS2020QN038).

## Conflict of Interest

The authors declare that the research was conducted in the absence of any commercial or financial relationships that could be construed as a potential conflict of interest.

## Publisher's Note

All claims expressed in this article are solely those of the authors and do not necessarily represent those of their affiliated organizations, or those of the publisher, the editors and the reviewers. Any product that may be evaluated in this article, or claim that may be made by its manufacturer, is not guaranteed or endorsed by the publisher.
